# A label-free optical whole-cell *Escherichia coli* biosensor for the detection of pyrethroid insecticide exposure

**DOI:** 10.1038/s41598-019-48907-6

**Published:** 2019-08-28

**Authors:** Pinpunya Riangrungroj, Candace Spier Bever, Bruce D. Hammock, Karen M. Polizzi

**Affiliations:** 1 Department of Life Sciences, Imperial College London, London, SW7 2AZ UK; 2 Imperial College Centre for Synthetic Biology, Imperial College London, London, SW7 2AZ UK; 3 Foodborne Toxin Detection and Prevention Unit, Western Regional Research Center, Agricultural Research Service, United States Department of Agriculture, Albany, CA 94710 USA; 4 Department of Entomology and UCD Cancer Center, University of California, Davis, California 95616 United States; 5 Department of Chemical Engineering, Imperial College London, London, SW7 2AZ UK

**Keywords:** Environmental biotechnology, Genetic circuit engineering, Synthetic biology

## Abstract

There is a growing need for low-cost, portable technologies for the detection of threats to the environment and human health. Here we propose a label-free, optical whole-cell *Escherichia coli* biosensor for the detection of 3-phenoxybenzoic acid (3-PBA), a biomarker for monitoring human exposure to synthetic pyrethroid insecticides. The biosensor functions like a competitive ELISA but uses whole-cells surface displaying an anti-3-PBA VHH as the detection element. When the engineered cells are mixed with 3-PBA-protein conjugate crosslinking that can be visually detected occurs. Free 3-PBA in samples competes with these crosslinks, leading to a detectable change in the output. The assay performance was improved by coloring the cells via expression of the purple-blue amilCP chromoprotein and the VHH expression level was reduced to obtain a limit of detection of 3 ng/mL. The optimized biosensor exhibited robust function in complex sample backgrounds such as synthetic urine and plasma. Furthermore, lyophilization enabled storage of biosensor cells for at least 90 days without loss of functionality. Our whole-cell biosensor is simple and low-cost and therefore has potential to be further developed as a screening tool for monitoring exposure to pyrethroids in low-resource environments.

## Introduction

Pyrethroids are a major class of insecticides, which are structurally modified from pyrethrins, the natural compounds found in the flowers of *Chrysanthemum cinerariaefolium*. These synthetic compounds have been extensively used worldwide for insect control in agricultural and household applications as they have a relatively low toxicity to mammals and a short half-life compared to DDT. Pyrethroids act as acute neurotoxins by blocking the voltage-gated sodium channels of insect nerve cells causing paralysis and death^1^. The widespread use of pyrethroids has raised concerns about negative impacts on the environment and human health. Environmental monitoring studies have shown high accumulation of these compounds in sediments^2,3^ raising the possibility that they can cause adverse impacts on organisms such as aquatic species^4,5^ and beneficial insects like honeybees and ground beetles^6,7^ within local ecosystems. Moreover, although these compounds are considered safe for humans, some studies have reported that acute exposure to high doses of pyrethroids can cause symptoms such as headache, nausea, and skin irritation^8–10^, while chronic exposure may severely affect human health, e.g., by suppressing the immune system^11,12^, disrupting the endocrine system^13^, and causing carcinogenesis according to *in vivo* studies in rat models^14,15^. Therefore, there are safety concerns regarding the health impacts of pyrethroid insecticide use.

Direct measurement of pyrethroid insecticide concentrations in human samples is difficult because of their rapid degradation and short half-lives (~6 h in urine and 2.5–12 h in plasma). Thus, the main method of detecting exposure relies on the measurement of 3-phenoxybenzoic acid (3-PBA), a common primary metabolite in the degradation of multiple insecticides^16,17^, as a biomarker. High performance liquid chromatography (HPLC) and gas chromatography–mass spectrometry (GC-MS) are the gold-standard assays for quantifying 3-PBA with limits of detection (LOD) of less than 0.5 ng/mL^16,18–20^. However, these assays are relatively time-consuming, expensive and lack portability for on-site application. Alternatively, cheaper and more rapid enzyme-linked immunosorbent assay (ELISA) methods have been developed. These are based on a competitive ELISA format where 3-PBA in the sample competes for antibody binding sites with an artificial antigen consisting of a 3-PBA-protein conjugate or a minor modification of this format. For example, colorimetric competitive ELISA assays using anti-3-PBA antibodies have been successfully demonstrated with a LOD between 0.1 and 1.94 ng/mL in urine and plasma samples^21–23^.

Moreover, it has been shown that single-domain camelid nanobodies (VHHs) can be substituted for the full-length conventional antibodies without impairing assay performance^24^. Since VHH antibodies are monomeric proteins expressed from a single gene, they are easier to express than conventional antibodies. Interestingly, using phages expressing multiple copies of the VHH as the detection element improved the LOD by an order of magnitude because the multi-valency of the phage increased the output signal^25^. More recently, Huo *et al*.^26^ reported the use of a VHH-alkaline phosphatase fusion protein (VHH-AP) for one-step 3-PBA detection. These findings highlight the significance of VHHs in developing assays for 3-PBA detection. However, even ELISA methods still require relatively specialist operators and sophisticated instruments for detection, which limits their field application.

Whole-cell bacterial biosensors are a low-cost, well-characterized and easy to manipulate alternative platform for detecting analytes. Whole-cell biosensors have been demonstrated as potential diagnostic tools for detecting a wide variety of compounds in clinical and environmental samples and have been engineered to produce a simple visible output such as pigments, luminescence or fluorescence signals^27,28^. Many studies have highlighted the possibility of applying bacterial biosensors for field detection because of their simplicity, low cost, and portability^29–32^.

**Figure 1:**
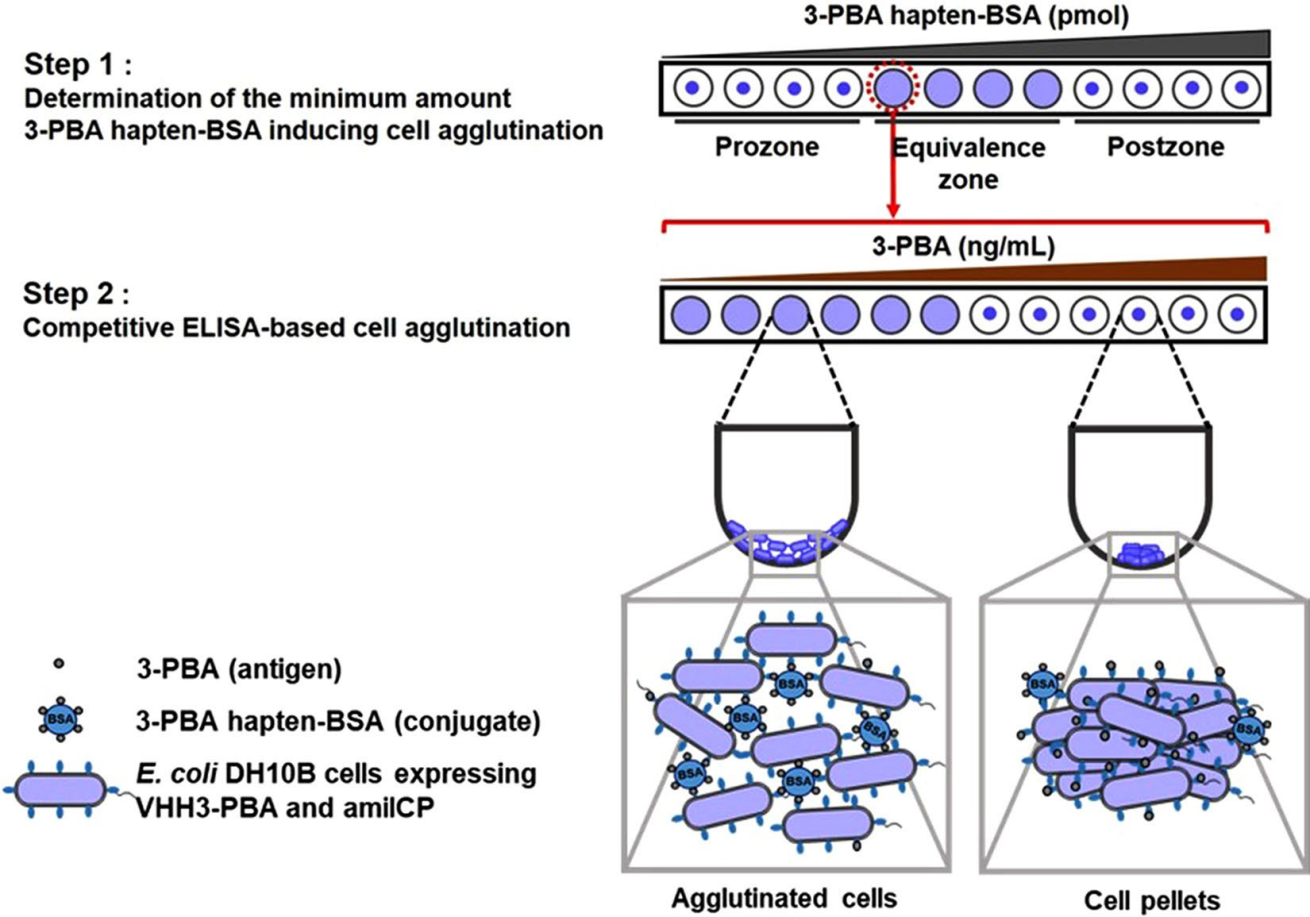
Schematic concept of agglutination based whole-cell *E. coli* biosensor for 3-PBA detection.

In this study, we demonstrate for the first time a label-free optical whole-cell *E. coli* biosensor for detecting the 3-PBA biomarker. In previous work we showed that cells expressing a VHH on their surface could be used as a modular detection platform for protein analytes. In an analogy to the latex agglutination test (LAT), the cells act as particles that are cross-linked by binding to multiple epitopes with the same antigen, resulting in agglutination^33^. However, the low molecular weight of 3-PBA makes such a direct detection format impossible. Instead, here we developed an assay that mimics a competitive ELISA by first using the 3-PBA hapten conjugated to bovine serum albumin (3-PBA hapten-BSA) to induce cell agglutination, followed by the application of samples containing free 3-PBA. The free 3-PBA acts as a binding competitor for the VHH and disrupts the agglutination reaction resulting in the formation of cell pellets (Fig. 1). This method is a simple and low-cost assay format that results in a visual output that is detectable by the naked eye, thus making it a promising tool for monitoring small-molecule biomarkers in the field as well as in resource-limited or rural environments.

## Results

### Establishing the competitive ELISA-based cell agglutination assay

As shown in Fig. 1, the *E. coli* biosensor concept is based on the principle of competitive ELISA, where interactions between 3-PBA hapten-BSA^22^ and surface-displayed VHHs are used to induce cell agglutination and free 3-PBA, e.g. in a sample, competes for VHH binding and disrupts agglutination leading to cell pellet formation. Thus, the presence of 3-PBA in a sample is transduced into an optical output in the form of a cell pellet.

To develop the biosensor, *E. coli* cells were transformed with a plasmid encoding a fusion protein of a β-intimin domain^34^ with an anti-3-PBA VHH^25^ under the control of an IPTG inducible promoter. The β-intimin domain acts as the anchoring motif at the outer membrane for transporting the VHH onto the cell surface. Originally, the anti-3-PBA VHH was isolated from clone 3P5ThC12 obtained from an immune library by phage display after immunization of a male alpaca with 3-PBA-thyroglobulin conjugate mixed with Freund’s incomplete adjuvant^25^. Cell surface expression of the anti-3-PBA VHH was first confirmed by flow cytometry and fluorescence microscopy analyses (Fig. SI1). Cell agglutination was achieved by using 3-PBA hapten-BSA, which was previously used as coating antigen in an immunoassay for pyrethroid metabolite detection^22^. The structure of this 3-PBA hapten-BSA conjugate is shown in Fig. SI2.

**Figure 2:**
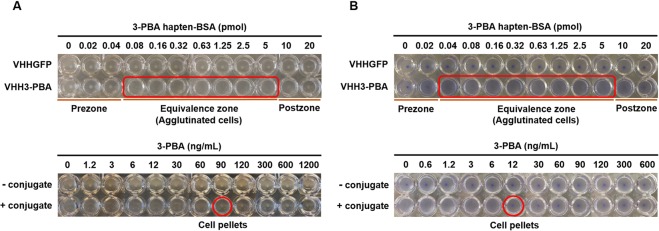
Agglutination assay for 3-PBA detection in the absence (**A**) and presence (**B**) of amilCP expression. Determination of 3-PBA hapten-BSA concentration necessary to induce agglutination of *E. coli* DH10B cells displaying anti-3-PBA VHH (VHH3-PBA, Top) and detection of 3-PBA in response to cell pellet formation at constant concentration of the conjugate (0.08 pmol with no amilCP co-expression and 0.04 pmol with amilCP coexpression) (Bottom). Red rectangles and circles represent regions in which cell agglutination occurred and the limit of detection, respectively. Pictures are a composite of photos that were taken every six wells per frame using a phone camera. This experiment was conducted in three biological replicates.

The appropriate amount of 3-PBA hapten-BSA conjugate needed to induce cell agglutination was determined by varying its concentrations in the presence of the cells (Fig. 2A, Top). Cells agglutinated in the presence of 3-PBA hapten-BSA in the range of 0.08–5 pmol of protein, but not at higher or lower concentrations, consistent with previous results that showed a prozone, postzone, and equivalence zone in the assay^33^. No cell agglutination was observed when the same concentrations of unconjugated BSA were used as a negative control (Fig. SI3). Moreover, incubating cells expressing the anti-GFP VHH with the 3-PBA hapten-BSA as a negative control did not lead to agglutination at any concentration tested.

Cell agglutination should be most sensitive to disruption from competition with free 3-PBA at the lowest concentration in the equivalence zone. Therefore, we chose this concentration to develop the 3-PBA competitive ELISA assay. Cells were incubated with mock samples of free 3-PBA in PBS at concentrations ranging from 1.2–1,200 ng/mL (in the original sample) in both the presence and absence of 0.08 pmol 3-PBA hapten-BSA (Fig. 2A, Bottom). In the absence of the protein conjugate, cells do not agglutinate, demonstrating that 3-PBA is monovalent, and in addition, is too small to induce cell crosslinking on its own. In the presence of the protein conjugate, cells agglutinate at low concentrations of free 3-PBA, but form pellets at concentrations above 90 ng/mL indicating that the competitive ELISA method can detect free 3-PBA above a certain threshold.

We next sought to improve the visual detection of the assay by changing the color of the *E*. coli cells through co-expression of the blue chromoprotein amilCP^35^. Cells were co-transformed with two separate plasmids encoding the inducible expression of the anti-3-PBA VHH and constitutive intracellular expression of amilCP, which led to a noticeable blue-purple color after 7 h of cell growth. The cells were then prepared and used in the agglutination assay as described previously. Figure 2B shows that the assay still functions as designed, but the limit of detection (both of the 3-PBA hapten-BSA and the free 3-PBA) has shifted to a lower concentration of analyte, likely as a result of the metabolic burden associated with amilCP expression leading to decreased expression of the VHH on the cell surface (Fig. SI4). As a result, the LOD was decreased to 12 ng/mL of 3-PBA (corresponding to 56 nM in the original sample), 7.5-fold lower than without amilCP co-expression (Fig. 2B, Bottom). Hence, co-expression of the amilCP protein not only improves the visualization of the assay, but also decreases the LOD.

### Improving the limit of detection by decreasing the VHH expression level

**Figure 3:**
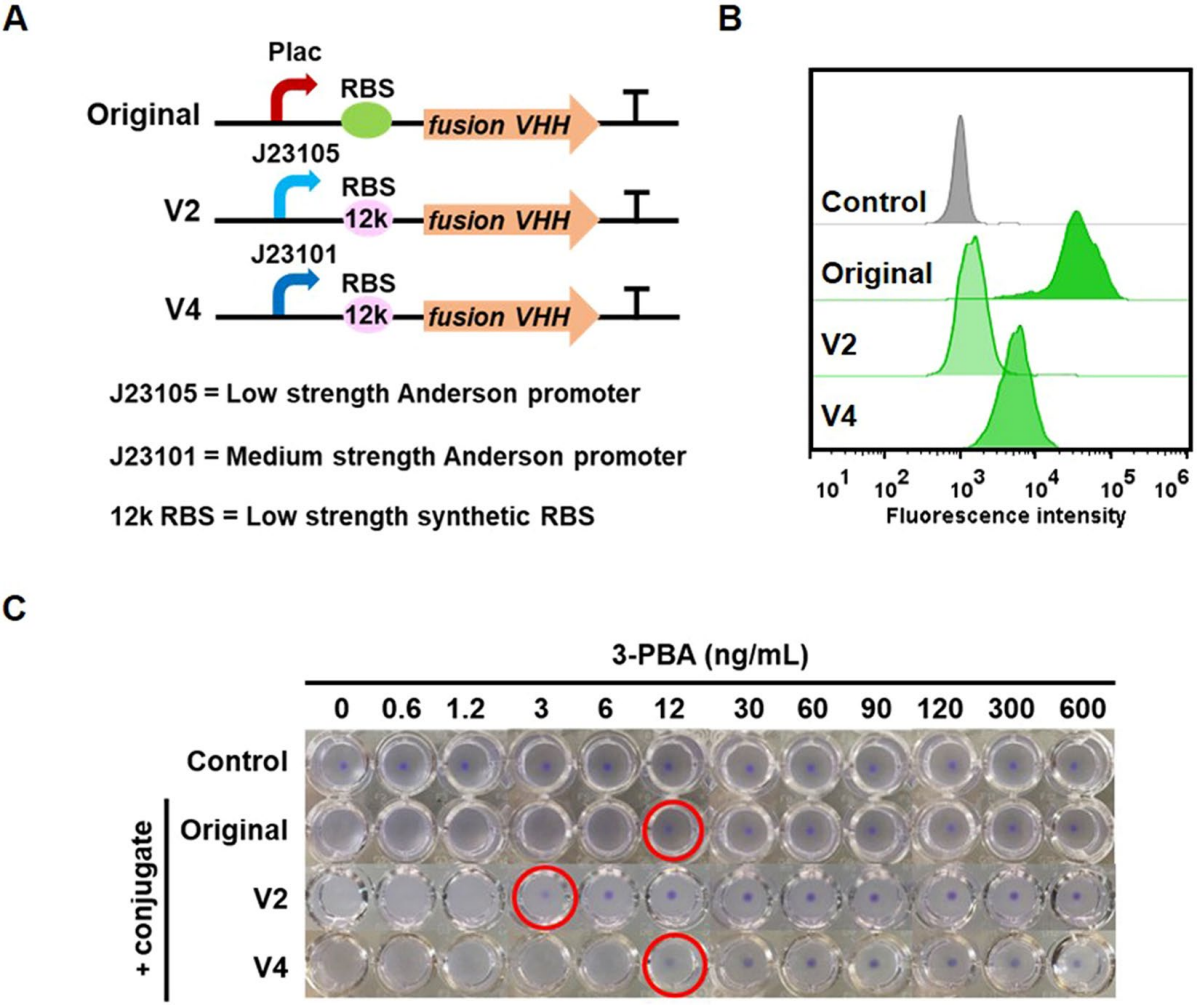
Lowering the limit of detection of 3-PBA by decreasing surface expression of the VHH. (**A**) Schematic of pNV3PBA plasmids containing promoters and RBSs with varied strengths upstream of the anti-3-PBA VHH fusion protein. (**B**) Histograms of the fluorescence intensity of cells carrying pNV3PBA probed with varied amounts of the anti-Myc-Tag (9B11) Mouse mAb (Alexa Fluor 488 conjugate). (**C**) Detection limit of 3-PBA concentration when the conjugate concentration was constant at 0.04 pmol. Red circles represent minimum concentration of 3-PBA inducing cell pellet formation. Pictures are a composite of photos that were taken every three wells per frame using a phone camera. This experiment was performed in three biological replicates.

Previous work, as well as the results in Fig. 2B, demonstrated that decreasing the surface expression of the VHH can be used as a mechanism for decreasing the LOD^33^, which is analogous to decreasing the concentration of coating antigen in a traditional ELISA. Therefore, we constructed plasmids containing different low-strength constitutive promoters and a synthetic RBS to fine-tune the VHH expression level (Fig. 3A) and transformed these into *E. coli* DH10B. Flow cytometry analysis was then used to quantify the number of surface-displayed VHHs by probing with the anti-Myc-Tag (9B11) Mouse mAb (Alexa Fluor 488 conjugate). Overall, cells transformed with the pNV3PBA V2 plasmid variant had the lowest surface expression of the VHH followed cells transformed with the pNV3PBA V4 plasmid variant and the original plasmid, respectively (Fig. 3B). The number of VHHs on the surface of cells transformed with the pNV3PBA V2 plasmid was approximately 15-fold lower than the original construct (Fig. SI4).

Cells transformed with the different plasmids were prepared and tested with varying concentrations of 3-PBA hapten-BSA to induce agglutination (Fig. 3C). In all cases, cell agglutination was induced upon addition of 0.04 pmol of conjugate. However, cells transformed with the pNV3PBA V2 plasmid had a narrower equivalence zone, suggesting that the number of VHHs on the surface has an effect on the agglutination reaction. Moreover, when agglutinated cells were incubated with samples of free 3-PBA of varying concentration, cells transformed with the pNV3PBA V2 plasmid formed a pellet upon addition of samples containing as little as 3 ng/mL of 3-PBA (corresponding to 14 nM, Fig. 3C). Thus, decreasing the surface expression of the VHH and co-expressing amilCP lowered the LOD 30-fold compared to the initial assay conditions.

### Evaluating the effect of the matrix using mock samples

**Figure 4:**
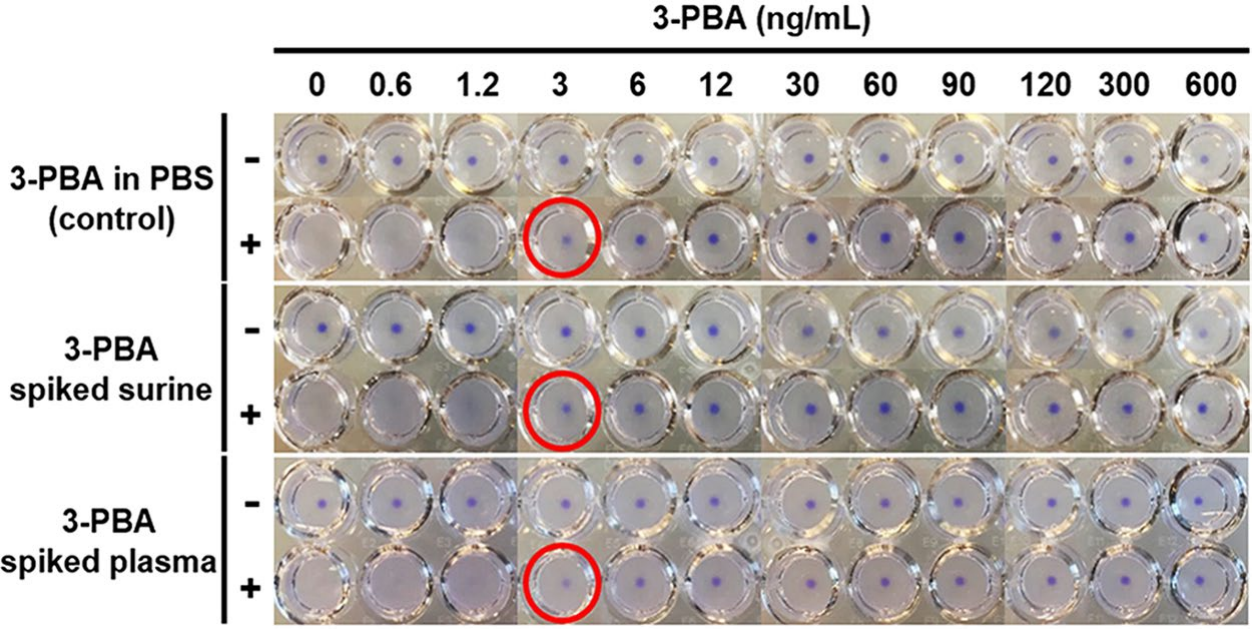
Cell agglutination assay in 3-PBA mock samples in urine or plasma. *E. coli* DH10B cells expressing anti-3-PBA VHH (V2 construct) and amilCP were mixed without (−) or with (+) 0.04 pmol 3-PBA hapten-BSA at various concentrations of 3-PBA spiked Surine (middle panel) and human plasma (bottom panel) and then serially diluted in PBS. Red circles represent minimum concentrations causing cell pellet formation. Pictures are a composite of photos that were taken six wells per frame using a phone camera. This experiment was conducted in three biological replicates.

As 3-PBA is a urinary or plasma biomarker, we next tested the effect of the assay matrix on the agglutination reaction by creating mock samples of known concentrations of 3-PBA spiked into Surine, a synthetic human urine, or fibrinogen-deficient plasma. The mock samples were then serially diluted and used in the assay with the cells transformed with the pNV3PBA V2 plasmid. As shown in Fig. 4, the LOD of the assay is 3 ng/mL of 3-PBA in both mock samples, which was the same LOD found in the control assay where 3-PBA was dissolved in PBS. These data suggest the assay can be applied to detect 3-PBA in human samples regardless of the presence of a complex matrix such as plasma or urine.

### Cell lyophilization for long-term stability

**Figure 5:**
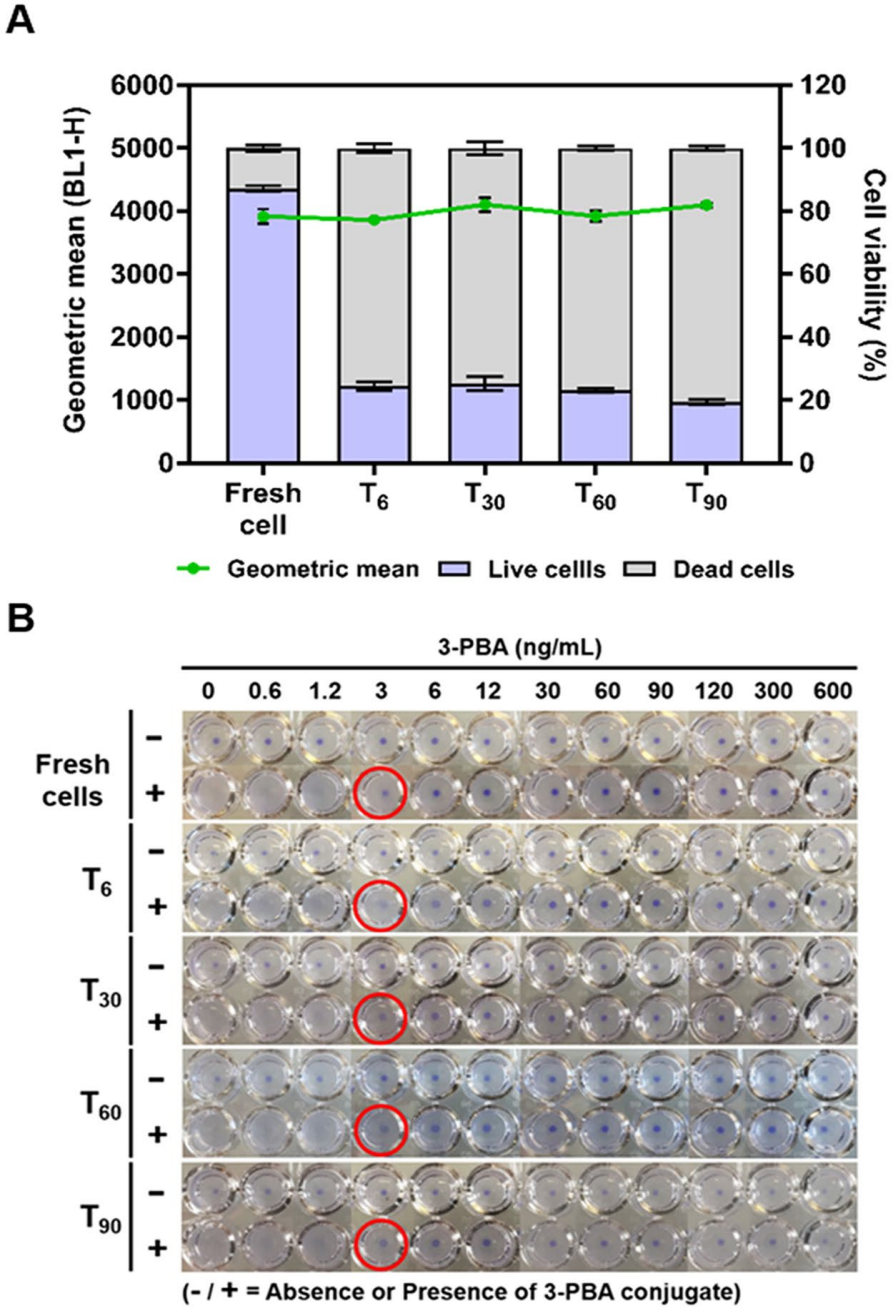
Lyophilization and storage of the whole-cell biosensor using *E. coli* DH10B cells expressing anti-3-PBA VHH (V2 construct) and amilCP. (**A**) Measurement of cell fluorescence intensity (VHH expression level) by probing cells with the anti-Myc-Tag (9B11) Mouse mAb (Alexa Fluor 488 conjugate) (green line) and cell viability using propidium iodide (PI) (bars) before (fresh cells) and after lyophilization with storage for 6, 30, 60 and 90 days. (**B**) Cell agglutination was induced with 0.04 pmol of the 3-PBA hapten-BSA conjugate and free 3-PBA concentrations were varied. Red circles represent the minimum concentrations forming cell pellets. Pictures are composites of photos that were taken every six wells per frame using a phone camera. This experiment was carried out in three technical replicates.

To facilitate the long-term storage of the whole-cell biosensor, and therefore, increase its portability for field studies, we investigated the effects of lyophilization of the cells on the assay performance. Cells transformed with the pNV3PBA V2 plasmid were prepared and then centrifuged and resuspended in 10% w/v sucrose solution as a lyoprotectant^36,37^ before being freeze-dried. To examine the effects of freeze drying on the cells, samples were rehydrated in PBS and flow cytometry was used to examine cell viability and the VHH surface expression. Samples were analyzed immediately prior to freeze-drying and after freeze-drying and storage for 6, 30, 60, or 90 days at 4 °C. Subjecting cells to lyophilization decreases their viability to around 20%, but the VHH is still retained on the cell surface at a similar expression level even after storage for up to 90 days at 4 °C (Fig. 5A). More importantly, the whole-cell biosensor still functions in the agglutination reaction with a consistent LOD across time (Fig. 5B). These data indicate that lyophilization can be a useful technique to facilitate field testing as it enables long-term storage of the whole-cell biosensor.

### Minimizing the analysis time

The initial workflow for the biosensor relied on a 16 h incubation for the development of the output signal. The limiting factor in detection is the formation of the cell pellet, which given the small size of *E. coli* takes >12 h to form when relying on gravity as the mechanism of settling. Therefore, we investigated whether the analysis time could be minimized by applying centrifugation to speed up the detection of cell pellet formation. The agglutination reaction was set up and incubated at room temperature for 10 min, then subsequently centrifuged at 250 × g for 15 min. In parallel, a duplicate assay was conducted and left to incubate for 16 h under static conditions as a control. The results show that the assay has similar performance under both conditions with an identical LOD, although the cell pellets from the assay subjected to centrifugation were less visually intense than those of the static assay (Fig. SI5). One possible explanation is that the cell agglutination reaction was incomplete after the 10-minute incubation time. Alternatively, it is possible that cells continue to multiply during the 16 h static incubation, leading to a larger visible cell mass that enables easier detection. Nevertheless, overall, the results suggest that the centrifugation technique could be an optional mechanism to shorten 3-PBA detection time to as little as 30 minutes, if desired.

## Discussion

Here we show the development of a novel approach for the detection of small molecule biomarkers using a label-free, optical whole-cell *E. coli* biosensor using the detection of 3-PBA as a proof-of-principle. The format of the assay is similar to a competitive ELISA but using whole-cells engineered to express a single-domain camelid VHH on their surface as a detection element. The cells are mixed with 3-PBA hapten-BSA, which induces cell agglutination. Free 3-PBA in samples can compete with the protein conjugate and prevent cell crosslinking leading to cell pellet formation. Therefore, the presence of 3-PBA can be visually detected by the naked eye. The assay presented is qualitative since the presence of 3-PBA above a certain threshold concentration will elicit a visual response, but the total number of cells in assay stays constant regardless of the presence of the analyte. To develop a quantitative assay, modification of the concept would be required so that agglutinated cells were retained and unagglutinated cells removed via a wash step so that a signal proportional to the analyte concentration would develop. Such an assay format could be achieved using a lateral flow strip or similar device.

After establishing the initial principle of the assay, we introduced a series of improvements. First, the visualization of the assay was enhanced by incorporating the expression of the amilCP chromoprotein as a dye in the cell to improve the visibility of the resulting cell pellets. Next, we reduced the LOD through modulating the surface expression of the VHH, which resulted in an optimized biosensor with a LOD of 3 ng/mL 3-PBA. The LOD is in a similar range as that reported previously for ELISA-based methods, which range from 0.01–2 ng/mL^21–23,25,26^. Finally, we showed that the analysis time can be reduced by introduction of a centrifugation step to precipitate the cells more rapidly. In resource-limited or rural field sites, this centrifugation step could plausibly be implemented using low-cost, hand-made centrifuges such as paperfuge^38^, egg-beater^39^, or salad spinner^40^.

In order for the whole-cell biosensor to be applied to real-world samples, the signal must be independent of the sample background. In the case of 3-PBA detection, because it is a metabolite and serves as a biomarker for pyrethroid exposure, samples will normally be either blood or urine, which can have interfering effects due to their complexity. Therefore, to test the robustness of our biosensor, we created mock samples where a known concentration of 3-PBA was dissolved in synthetic urine or plasma and then subjected them to the assay procedure. We obtained the same results with respect to LOD regardless of the matrix in which the sample was prepared, suggesting samples in urine or blood will be amenable to measurement using our whole-cell biosensor. In addition, due to the high sensitivity of this assay, there was no need for chemical extraction, which has impacted the performance of previous antibody-based methods^23^.

To facilitate portability for field testing, we showed that cells could be lyophilized and stored without loss of functionality for up to 90 days. This will also enable the whole-cell biosensor to be manufactured in batches in advance in a central facility, stored and distributed when needed, which will allow for quality control over batches that should ensure reproducibility of materials. Moreover, although a large proportion of the population becomes non-viable during lyophilization, the LOD and the VHH surface expression in the dead cells remain unaltered. This opens up the possibility that cells could be intentionally inactivated, either via manipulating the lyophilization conditions or via genetic mechanisms, for example, bacterial ghost technology^41–43^. Overall, our whole-cell *E. coli* biosensor meets many of the criteria for feasible use in point-of-care testing or field studies. Future work could focus on further developing this concept to create a diagnostic tool to monitor not only the 3-PBA biomarker but also other environmental contaminants for which VHHs have already been isolated, such as those discussed in Bever *et al*.^24^ and clinically relevant biomarkers in human samples.

## Materials and Methods

### Bacterial cells and plasmids

*E. coli* strains DH5α (New England Biolabs, UK) and DH10B (Thermo Fisher Scientific, UK) were used for cloning and VHH expression, respectively. The pNV3PBA plasmid for *E. coli* surface display was created using the pNVgfp plasmid^34^ as a backbone vector and replacing the anti-GFP VHH sequence with that of the anti-3-PBA VHH amplified from the pComb3xSS plasmid^25^ using Gibson DNA assembly^44^. The pNV3PBA V2 and pNV3PBA V4 plasmids were constructed by replacing the lac-inducible promoter and Shine-Dalgarno sequences with the J23101 or J23105 constitutive promoter^45^ and a synthetic ribosome binding site (RBS) created using the RBS calculator^46^ The pAmilCP_J104 plasmid was created for increasing the visibility of *E. coli* cells by assembling the J23104 constitutive promoter, the B0034 RBS, and the amilCP chromoprotein coding sequence in the pSB1C3 vector backbone from the iGEM Registry of Standard Biological parts (partsregistry.org). The full sequences of all plasmids can be found in the Supplementary Information.

### Bacterial cell cultivation

A single colony of *E. coli* DH10B cells transformed with the pNV3PBA and pAmilCP_J104 plasmids was grown overnight in 10 mL of LB broth (VWR, UK) supplemented with 100 µg/mL ampicillin and 34 µg/mL chloramphenicol (Sigma-Aldrich, UK) at 37 °C with shaking at 250 rpm. On the following day, cells were diluted 1:500 in 100 mL of fresh medium and VHH expression was induced with a final concentration of 1 mM of IPTG. Cells were incubated at 37 °C with shaking at 250 rpm for 7 h, harvested by centrifugation at 3200 × g for 10 min at room temperature (Eppendorf 5810 R, Germany), washed twice in sterile-filtered 1x phosphate buffered saline pH 7.4 (PBS) and resuspended to a final OD_600_ of 1 (∼1 × 10^9^ cells) in PBS before use in the assay. The pNV3PBA V2 and pNV3PBA V4 plasmids contain constitutive promoters that do not require IPTG for induction. Here, the overnight culture was diluted 1:500 in fresh medium and grown at 37 °C with shaking at 250 rpm for 7 h, followed by the protocol described above for cell preparation.

### Competitive ELISA cell agglutination assay

The 3-PBA-BSA conjugate (cAg01) was synthesized as previously described^22^. To develop the competitive ELISA format, a defined amount of 3-PBA hapten-BSA was mixed in equal volumes with a solution containing various concentrations (0–300 ng/mL) of free 3-PBA (Alfa Aesar, UK) in PBS. 200 µL of cell suspension (∼2 × 10^8^ cells) was mixed with 40 μL of the analyte solutions in a clear round-bottom 96-well plate (Costar, USA) and pipetted up and down to mix thoroughly. The reaction was then incubated for 16 h at room temperature under static conditions or centrifuged at 250 × g for 15 min.

### Mock sample preparation

3-PBA was spiked into Surine Negative Control Urine (Sigma-Aldrich, USA) and fibrinogen-deficient plasma (Sekisui Diagnostics GmbH, Germany) at various concentrations to create mock samples, which were used in the assay as described above.

### Flow cytometry analysis (FACS)

To measure surface expression of the VHH, 100 μL of cell suspension (∼5 × 10^7^ cells) was incubated with 5 µL of 50 μg/mL anti-Myc-Tag (9B11) Mouse mAb (Alexa Fluor 488 conjugate) (Cell Signaling Technology, UK) at 4 °C in the dark for 30 min. The cells were washed twice and resuspended in 50 μL PBS. To test for cell viability, 1 µL of 1 mg/mL propidium iodide solution (Sigma-Aldrich, USA) was added. Cells were diluted 200-fold in PBS and analyzed using an Attune NxT flow cytometer (Thermo Fisher Scientific, USA) with collection of at least 100,000 events. Uninduced cells were used as a negative control and for gating. Data analysis was carried out using the FlowJo vX.0.7 software (FlowJo, LLC, USA).

For quantification of VHH expression, the same protocol was used, but the amount of the labelling antibody was varied (0–10 pmol) and the correlation curve between geometric means and the antibody amounts was fitted using a non-linear least squares regression model in Prism 7 software (GraphPad, USA) to estimate the concentration at which binding of the anti-Myc-Tag antibody was saturated.

### Microscopy analysis

Glass slides were prepared with 2 μL of labelled cells and examined under a Nikon Eclipse Ti inverted microscope using a 60x objective and a 100 ms exposure time with a 488 nm laser as an excitation light for green fluorescence detection (Nikon Instruments Europe, Netherland). Cells with an empty plasmid were used as a negative control for autofluorescence.

### Cell lyophilization

Cells expressing the VHH were washed twice and resuspended in sterile lyophilization solution containing 10% w/v sucrose (Sigma-Aldrich, USA) in distilled water (DW) at an OD_600_ of ∼15. One mL aliquots were distributed into autoclaved glass vials and frozen at −80 °C for 16 h. Frozen samples were freeze-dried at a condenser temperature of −100 °C and a pressure of 45 mTor for 24 h in a BenchTop Pro with Omnitronics lyophilizer, (SP Scientific, USA) and then stored at 4 °C for 6, 30, 60, or 90 days. Lyophilized cells were rehydrated with 4 mL of PBS at room temperature for 2 h before use in FACS analysis or for competitive ELISA agglutination assays as described above.

## Supplementary information

Supplementary Information

## Data Availability

All data generated or analyzed during this study are included in this published article (and its Supplementary Information files) and can be used without restriction.
