# Supplementary material for: A label-free optical whole-cell Escherichia coli biosensor for the detection of pyrethroid insecticide exposure
Source: Sci Rep. 2019 Aug 28;9:12466. doi: 10.1038/s41598-019-48907-6 (PMC6713742; doi:10.1038/s41598-019-48907-6)
Supplement: Supplementary file 1 — Supplementary Information [file 41598_2019_48907_MOESM1_ESM.pdf]

Supplementary Information

A label-free optical whole-cell *Escherichia coli* biosensor for the detection of pyrethroid insecticide exposure

Pinpunya Riangrungroj[1,2], Candace Spier Bever[3], Bruce D. Hammock[4], Karen M. Polizzi[2,5,*]

[1] *Department of Life Sciences, Imperial College London, London, SW7 2AZ, UK*

[2] *Imperial College Centre for Synthetic Biology, Imperial College London, London, SW7 2AZ, UK*

[3] *Foodborne Toxin Detection and Prevention Unit, Western Regional Research Center, Agricultural Research Service, United States Department of Agriculture, Albany, CA 94710, USA*

[4] *Department of Entomology and UCD Cancer Center, University of California, Davis, California 95616, United States*

[5] *Department of Chemical Engineering, Imperial College London, London, SW7 2AZ, UK*

* Corresponding author

E-mail address: k.polizzi@imperial.ac.uk (K.M. Polizzi)

Phone: (44) 02075942851

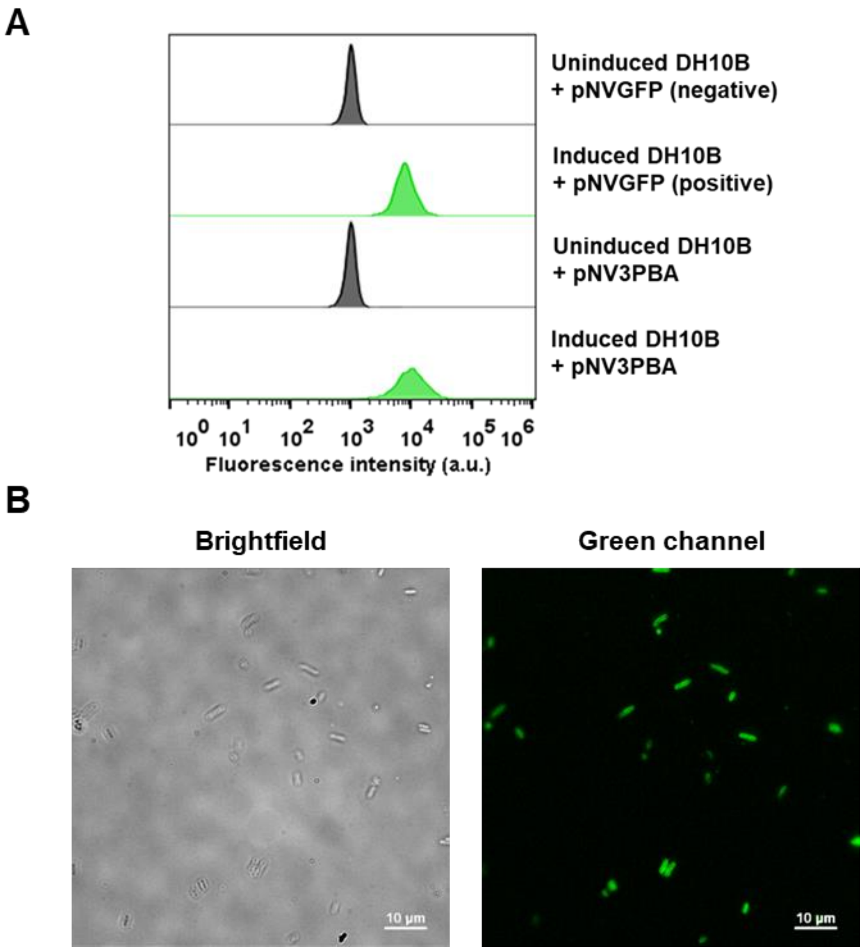

Figure SI1. The cell surface expression of VHH3-PBA. (A). Flow cytometry analysis of *E. coli* DH10B cells displaying VHH3-PBA on the surface. Cells labelled with anti-Myc-Tag (9B11) Mouse mAb (Alexa Fluor® 488 conjugate) were excited by a 488-nm blue laser and detecting through a 530/30 band-pass filter (BL1-H). Data were analyzed using the FlowJo software tool. (B). The brightfield and green fluorescent images of the antibody-bound cells were taken using a 60x objective at 100 ms exposure time using a laser with 488 nm excitation.

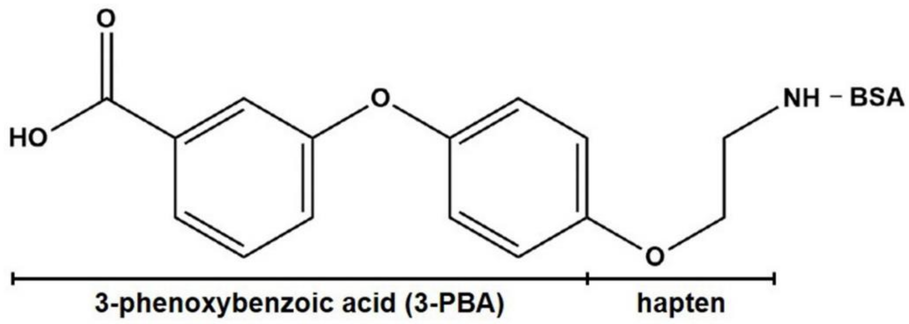

Figure SI2. Structure of 3-PBA hapten-BSA (cAg01). Hapten linker was attached to the 4-position of the distal phenyl group of 3-PBA and primary amine of lysine residue in BSA molecule.[1]

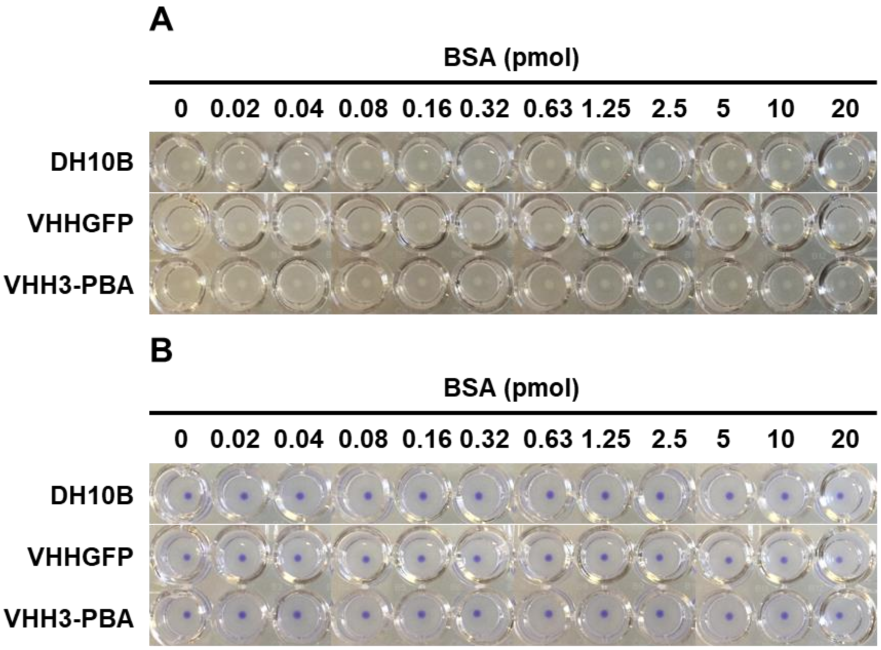

Figure SI3. BSA has no effect on the cell agglutination reaction. *E. coli* DH10B cells displaying VHH3-PBA in the absence (A) and presence (B) of amilCP were mixed with BSA at concentrations equivalent to those of 3-PBA hapten-BSA in the experiments. The reactions were statically incubated at room temperature for 16 h before observation. Pictures are a composite of photos that were taken every three or six wells per frame using a phone camera. This experiment was carried out in three technical replicates.

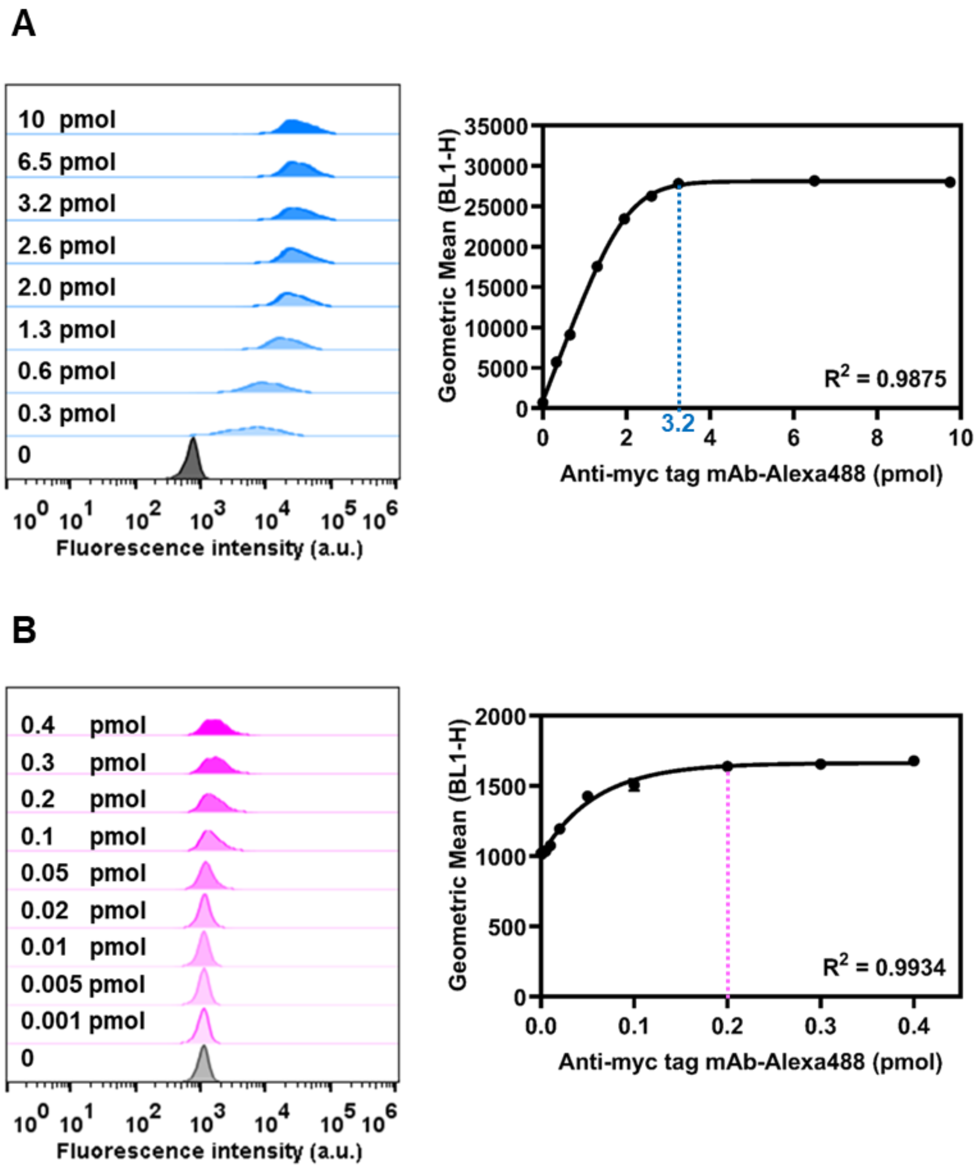

Figure SI4. Quantification of VHH3-PBA displayed on the surface of *E. coli* DH10B carrying pNV3PBA original (A) and V2 constructs (B) in the presence of amilCP. The induced cells were labelled with various amounts of the anti-Myc-Tag (9B11) Mouse mAb (Alexa Fluor® 488 conjugate) and then analyzed by flow cytometry. From left to right**,** histograms of the cell fluorescence intensity and the plotted curve of geometric means against the labelling antibody amounts was fitted using a non-linear least squares regression model in Prism 7 software (GraphPad). Dotted lines represent the initial point of the plateau assuming full occupancy of the labelled VHH. Mean and standard deviation were analyzed from three biological triplicates.

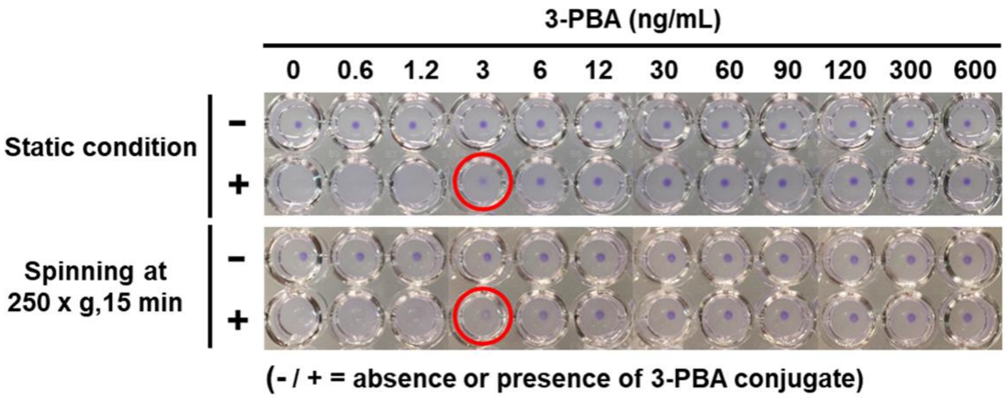

Figure SI5. Minimizing the analysis time by centrifugation. *E. coli* DH10B cells expressing VHH3-PBA (V2 construct) and amilCP were mixed without (-) or with (+) 0.04 pmol of 3-PBA hapten-BSA and various concentrations of 3-PBA diluted in PBS and statically incubated at room temperature for 10 min prior to spinning at 250 x g for 15 min. The standard analysis procedure with 16 h static incubation at room temperature was included as a control (top rows). Red circles represent minimum concentrations causing cell pellet. Pictures are a composite of photos that were taken six wells per frame using a phone camera.

Sequences of the plasmids used in this study

pNV3PBA

This plasmid was constructed using pNVgfp as a template[2] with the replacement of VHHgfp with VHH3PBA, amplified from pComb3xSS.[3]

lacI-plac promoter-NV fusion (IntiminEHEC-**E-tag**-**VHH3PBA**-**myc-tag**)-CmR-pBR322 ori

ACCCGACACCATCGAATGGCGCAAAACCTTTCGCGGTATGGCATGATAGCGCCCGGAAGAGAGTCAATTCAGGGTGGTGAATGTGAAACCAGTAACG
TTATACGATGTCGCAGAGTATGCCGGTGTCTCTTATCAGACCGTTTCCCGCGTGGTGAACCAGGCCAGCCACGTTTCTGCGAAAACGCGGGAAAAAG
TGGAAGCGGCGATGGCGGAGCTGAATTACATTCCCAACCGCGTGGCACAACAACTGGCGGGCAAACAGTCGTTGCTGATTGGCGTTGCCACCTCCAG
TCTGGCCCTGCACGCGCCGTCGCAAATTGTCGCGGCGATTAAATCTCGCGCCGATCAACTGGGTGCCAGCGTGGTGGTGTCGATGGTAGAACGAAGC
GGCGTCGAAGCCTGTAAAGCGGCGGTGCACAATCTTCTCGCGCAACGCGTCAGTGGGCTGATCATTAACTATCCGCTGGATGACCAGGATGCCATTG
CTGTGGAAGCTGCCTGCACTAATGTTCCGGCGTTATTTCTTGATGTCTCTGACCAGACACCCATCAACAGTATTATTTTCTCCCATGAAGACGGTAC
GCGACTGGGCGTGGAGCATCTGGTCGCATTGGGTCACCAGCAAATCGCGCTGTTAGCGGGCCCATTAAGTTCTGTCTCGGCGCGTCTGCGTCTGGCT
GGCTGGCATAAATATCTCACTCGCAATCAAATTCAGCCGATAGCGGAACGGGAAGGCGACTGGAGTGCCATGTCCGGTTTTCAACAAACCATGCAAA
TGCTGAATGAGGGCATCGTTCCCACTGCGATGCTGGTTGCCAACGATCAGATGGCGCTGGGCGCAATGCGCGCCATTACCGAGTCCGGGCTGCGCGT
TGGTGCGGACATCTCGGTAGTGGGATACGACGATACCGAAGACAGCTCATGTTATATCCCGCCGTTAACCACCATCAAACAGGATTTTCGCCTGCTG
GGGCAAACCAGCGTGGACCGCTTGCTGCAACTCTCTCAGGGCCAGGCGGTGAAGGGCAATCAGCTGTTGCCCGTCTCACTGGTGAAAAGAAAAACCA
CCCTGGCGCCCAATACGCAAACCGCCTCTCCCCGCGCGTTGGCCGATTCATTAATGCAGCTGGCACGACAGGTTTCCCGACTGGAAAGCGGGCAGTG
AGCGGTACCCGATAAAAGCGGCTTCCTGACAGGAGGCCGTTTTGTTTTGCAGCCCACCTCAACGCAATTAATGTGAGTTAGCTCACTCATTAGGCAC
CCCAGGCTTTACACTTTATGCTTCCGGCTCGTATGTTGTGTGGAATTGTGAGCGGATAACAATTTCACACAGGAAACAGCTATGACCATGATTACGA
ATTTCTAGATAACGAGGGCAAATCATGATTACTCATGGTTGTTATACCCGGACCCGGCACAAGCATAAGCTAAAAAAAACATTGATTATGCTTAGTG
CTGGTTTAGGATTGTTTTTTTATGTTAATCAGAATTCATTTGCAAATGGTGAAAATTATTTTAAATTGGGTTCGGATTCAAAACTGTTAACTCATGA
TAGCTATCAGAATCGCCTTTTTTATACGTTGAAAACTGGTGAAACTGTTGCCGATCTTTCTAAATCGCAAGATATTAATTTATCGACGATTTGGTCG
TTGAATAAGCATTTATACAGTTCTGAAAGCGAAATGATGAAGGCCGCGCCTGGTCAGCAGATCATTTTGCCACTCAAAAAACTTCCCTTTGAATACA
GTGCACTACCACTTTTAGGTTCGGCACCTCTTGTTGCTGCAGGTGGTGTTGCTGGTCACACGAATAAACTGACTAAAATGTCCCCGGACGTGACCAA
AAGCAACATGACCGATGACAAGGCATTAAATTATGCGGCACAACAGGCGGCGAGTCTCGGTAGCCAGCTTCAGTCGCGATCTCTGAACGGCGATTAC
GCGAAAGATACCGCTCTTGGTATCGCTGGTAACCAGGCTTCGTCACAGTTGCAGGCCTGGTTACAACATTATGGAACGGCAGAGGTTAATCTGCAGA
GTGGTAATAACTTTGACGGTAGTTCACTGGACTTCTTATTACCGTTCTATGATTCCGAAAAAATGCTGGCATTTGGTCAGGTCGGAGCGCGTTACAT
TGACTCCCGCTTTACGGCAAATTTAGGTGCGGGTCAGCGTTTTTTCCTTCCTGCAAACATGTTGGGCTATAACGTCTTCATTGATCAGGATTTTTCT
GGTGATAATACCCGTTTAGGTATTGGTGGCGAATACTGGCGAGACTATTTCAAAAGTAGCGTTAACGGCTATTTCCGCATGAGCGGCTGGCATGAGT
CATACAATAAGAAAGACTATGATGAGCGCCCAGCAAATGGCTTCGATATCCGTTTTAATGGCTATCTACCGTCATATCCGGCATTAGGCGCCAAGCT
GATATATGAGCAGTATTATGGTGATAATGTTGCTTTGTTTAATTCTGATAAGCTGCAGTCGAATCCTGGTGCGGCGACCGTTGGTGTAAACTATACT
CCGATTCCTCTGGTGACGATGGGGATCGATTACCGTCATGGTACGGGTAATGAAAATGATCTCCTTTACTCAATGCAGTTCCGTTATCAGTTTGATA
AATCGTGGTCTCAGCAAATTGAACCACAGTATGTTAACGAGTTAAGAACATTATCAGGCAGCCGTTACGATCTGGTTCAGCGTAATAACAATATTAT
TCTGGAGTACAAGAAGCAGGATATTCTTTCTCTGAATATTCCGCATGATATTAATGGTACTGAACACAGTACGCAGAAGATTCAGTTGATCGTTAAG
AGCAAATACGGTCTGGATCGTATCGTCTGGGATGATAGTGCATTACGCAGTCAGGGCGGTCAGATTCAGCATAGCGGAAGCCAAAGCGCACAAGACT
ACCAGGCTATTTTGCCTGCTTATGTGCAAGGTGGCAGCAATATTTATAAAGTGACGGCTCGCGCCTATGACCGTAATGGCAATAGCTCTAACAATGT
ACAGCTTACTATTACCGTTCTGTCGAATGGTCAAGTTGTCGACCAGGTTGGGGTAACGGACTTTACGGCGGATAAGACTTCGGCTAAAGCGGATAAC
GCCGATACCATTACTTATACCGCGACGGTGAAAAAGAATGGGGTAGCTCAGGCTAATGTCCCTGTTTCATTTAATATTGTTTCAGGAACTGCAACTC
TTGGGGCAAATAGTGCCAAAACGGATGCTAACGGTAAGGCAACCGTAACGTTGAAGTCGAGTACGCCAGGACAGGTCGTCGTGTCTGCTAAAACCGC
GGAGATGACTTCAGCACTTAATGCCAGTGCGGTTATATTTTTTGAT**GGTGCGCCGGTGCCGTATCCGGATCCGCTGGAACCGGCC**CAGCCGGCCCA
GGTGCAGCTCGTGGAGTCTGGGGGAGGCTTGGTGCAGCCTGGGGGGTCTCTGAGACTCTCCTGCGCAGCCTCTGGATTTAGT
TTGGAATATTATGGCATCGGCTGGTTCCGCCAGGCCCCAGGGAAGGAGCGTGAGGGGGTCGCATGTATTAGTAGTAGTGGTG

GACGCACAAACATTAGAGAAGGGCGATTCACCATCTCCAGAGACAACGCCGAGAACACGGTGTATCTGCAACTGAACAGCCTG
AAACCTGAGGACACAGCCATTTATTACTGTGCAGCAGCTCCCCAATACATTTGCGGTACTTACCGGTACACGGCGTTTCGTACC
TGGGGCCAGGGGACCCAGGTCACCGTCTCCTCGGAACCCAAGACACCAAAACCACAAGACGGCCAGGCCGGCCAGGCGGCCG
CC**GAACAAAAACTCATCTCAGAAGAGGAT**GCAGCTGCATAAAAGCTTGACCTGTGAAGTGAAAAATGGCGCACATTGTGCGACATTTTTTTTGTCTG
CCGTTTACCGCTACTGCGTCACGGATCCCCACGCGCCCTGTAGCGGCGCATTAAGCGCGGCGGGTGTGGTGGTTACGCGCAGCGTGACCGCTACACT
TGCCAGCGCCCTAGCGCCCGCTCCTTTCGCTTTCTTCCCTTCCTTTCTCGCCACGTTCGCCGGCTTTCCCCGTCAAGCTCTAAATCGGGGCATCCCT
TTAGGGTTCCGATTTAGTGCTTTACGGCACCTCGACCCCAAAAAACTTGATTAGGGTGATGGTTCACGTAGTGGGCCATCGCCCTGATAGACGGTTT
TTCGCCCTTTGACGTTGGAGTCCACGTTCTTTAATAGTGGACTCTTGTTCCAAACTGGAACAACACTCAACCCTATCTCGGTCTATTCTTTTGATTT
ATAAGGGATTTTGCCGATTTCGGCCTATTGGTTAAAAAATGAGCTGATTTAACAAAAATTTAACGCGAATTTTAACAAAATATTAACGTTTACAATT
TCAGGTGGCACTTTTCGGGGAAATGTGCGCGGAACCCCTATTTGTTTATTTTTCTAAATACATTCAAATATGTATCCGCTCATGTCGAGACGTTGGG
TGAGGTTCCAACTTTCACCATAATGAAATAAGATCACTACCGGGCGTATTTTTTGAGTTATCGAGATTTTCAGGAGCTAAGGAAGCTAAAATGGAGA
AAAAAATCACTGGATATACCACCGTTGATATATCCCAATGGCATCGTAAAGAACATTTTGAGGCATTTCAGTCAGTTGCTCAATGTACCTATAACCA
GACCGTTCAGCTGGATATTACGGCCTTTTTAAAGACCGTAAAGAAAAATAAGCACAAGTTTTATCCGGCCTTTATTCACATTCTTGCCCGCCTGATG
AATGCTCATCCGGAGTTCCGTATGGCAATGAAAGACGGTGAGCTGGTGATATGGGATAGTGTTCACCCTTGTTACACCGTTTTCCATGAGCAAACTG
AAACGTTTTCATCGCTCTGGAGTGAATACCACGACGATTTCCGGCAGTTTCTACACATATATTCGCAAGATGTGGCGTGTTACGGTGAAAACCTGGC
CTATTTCCCTAAAGGGTTTATTGAGAATATGTTTTTCGTCTCAGCCAATCCCTGGGTGAGTTTCACCAGTTTTGATTTAAACGTGGCCAATATGGAC
AACTTCTTCGCCCCCGTTTTCACCATGGGCAAATATTATACGCAAGGCGACAAGGTGCTGATGCCGCTGGCGATTCAGGTTCATCATGCCGTCTGTG
ATGGCTTCCATGTCGGCAGAATGCTTAATGAATTACAACAGTACTGCGATGAGTGGCAGGGCGGGGCGTAATTTTTTTAAGGCAGTTATTGGTGCCC

TTAAACGCCTGGTGCTACGCCTGAATAAGTGATAATAAGCGGATGAATGGCAGAAATTCGAAAGCAAATTCGACCCGGTCGTCGGTTCAGGGCAGGG
TCGTTAAATAGCCGCTTATGTCTATTGCTGGTTTACCGGTTTATTGACTACCGGAAGCAGTGTGACCGTGTGCTTCTCAAATGCCTGAGGCCAGTTT
GCTCAGGCTCTCCCCGTGGAGGTAATAATTGCTCGACATGACCAAAATCCCTTAACGTGAGTTTTCGTTCCACTGAGCGTCAGACCCCGTAGAAAAG
ATCAAAGGATCTTCTTGAGATCCTTTTTTTCTGCGCGTAATCTGCTGCTTGCAAACAAAAAAACCACCGCTACCAGCGGTGGTTTGTTTGCCGGATC
AAGAGCTACCAACTCTTTTTCCGAAGGTAACTGGCTTCAGCAGAGCGCAGATACCAAATACTGTCCTTCTAGTGTAGCCGTAGTTAGGCCACCACTT
CAAGAACTCTGTAGCACCGCCTACATACCTCGCTCTGCTAATCCTGTTACCAGTGGCTGCTGCCAGTGGCGATAAGTCGTGTCTTACCGGGTTGGAC
TCAAGACGATAGTTACCGGATAAGGCGCAGCGGTCGGGCTGAACGGGGGGTTCGTGCACACAGCCCAGCTTGGAGCGAACGACCTACACCGAACTGA
GATACCTACAGCGTGAGCTATGAGAAAGCGCCACGCTTCCCGAAGGGAGAAAGGCGGACAGGTATCCGGTAAGCGGCAGGGTCGGAACAGGAGAGCG
CACGAGGGAGCTTCCAGGGGGAAACGCCTGGTATCTTTATAGTCCTGTCGGGTTTCGCCACCTCTGACTTGAGCGTCGATTTTTGTGATGCTCGTCA
GGGGGGCGGAGCCTATGGAAAAACGCCAGCAACGCGGCCTTTTTACGGTTCCTGGCCTTTTGCTGGCCTTTTGCTCACATG

pNV3PBA V2 and V4

The original promoter and RBS in the pNV3PBA were replaced with promoters from Anderson promoter collection[4] and RBS using RBS calculator developed by Salis [5] for constitutive expression of VHH3PBA. These BioBrick sequences can be found in Table S1.

Table S1. Sequences of synthetic promoters and RBSs.

Construct	Sequence
V2 (J23105 promoter-RBS(12k))	TTTACGGCTAGCTCAGTCCTAGGTACTATGCTAGCAAAGGAAATCTA
V4 (J23101 promoter-RBS(12k))	TTTACAGCTAGCTCAGTCCTAGGTATTATGCTAGCAAAGGAAATCTA

pAmilCP

J23104 promoter-RBS(B0034)T-amilCP-B0010-rrnB T2 terminator-AmpR-p15A ori

GATAACATCAAATTTGACAGCTAGCTCAGTCCTAGGTATTGTGCTAGCTACTAGAGAAAGAGGAGAAATACTAGATGAGTGTGATCGCTAAACAAAT
GACCTACAAGGTTTATATGTCAGGCACGGTCAATGGACACTACTTTGAGGTCGAAGGCGATGGAAAAGGTAAGCCCTACGAGGGGGAGCAGACGGTA
AAGCTCACTGTCACCAAGGGCGGACCTCTGCCATTTGCTTGGGATATTTTATCACCACAGTGTCAGTACGGAAGCATACCATTCACCAAGTACCCTG
AAGACATCCCTGACTATGTAAAGCAGTCATTCCCGGAGGGCTATACATGGGAGAGGATCATGAACTTTGAAGATGGTGCAGTGTGTACTGTCAGCAA
TGATTCCAGCATCCAAGGCAACTGTTTCATCTACCATGTCAAGTTCTCTGGTTTGAACTTTCCTCCCAATGGACCTGTCATGCAGAAGAAGACACAG
GGCTGGGAACCCAACACTGAGCGTCTCTTTGCACGAGATGGAATGCTGCTAGGAAACAACTTTATGGCTCTGAAGTTAGAAGGAGGCGGTCACTATT
TGTGTGAATTTAAAACTACTTACAAGGCAAAGAAGCCTGTGAAGATGCCAGGGTATCACTATGTTGACCGCAAACTGGATGTAACCAATCACAACAA
GGATTACACTTCGGTTGAGCAGTGTGAAATTTCCATTGCACGCAAACCTGTGGTCGCCTAATAATACTAGTAGCGGCCGCTGCAGTGGCGGATGAGA
GAAGATTTTCAGCCTGATACAGATTAAATCAGAACGCAGAAGCGGTCTGATAAAACAGAATTTGCCTGGCGGCAGTAGCGCGGTGGTCCCACCTGAC
CCCATGCCGAACTCAGAAGTGAAACGCCGTAGCGCCGATGGTAGTGTGGGGTCTCCCCATGCGAGAGTAGGGAACTGCCAGGCATCAAATAAAACGA
AAGGCTCAGTCGAAAGACTGGGCCTTTCGTTTTATCTGTTGTTTGTCGGTGAACGCTCTCCTGAGTAGGACAAATCCGCCGGGAGCGGATTTGAACG
TTGCGAAGCAACGGCCCGGAGGGTGGCGGGCAGGACGCCCGCCATAAACTGCCAGGCATCAAATTAAGCAGAAGGCCATCCTGACGGATGGCCTTTT
TGCGTTTCTACAAACTCTTTTTGTTTATTTCATTCAAATATGTATCCGCTCATGAGACAATAACCCTGATAAATGCTTCAATAATATTGAAAAAGGA
AGAGTATGAGTATTCAACATTTCCGTGTCGCCCTTATTCCCTTTTTTGCGGCATTTTGCCTTCCTGTTTTTGCTCACCCAGAAACGCTGGTGAAAGT
AAAAGATGCTGAAGATCAGTTGGGTGCACGAGTGGGTTACATCGAACTGGATCTCAACAGCGGTAAGATCCTTGAGAGTTTTCGCCCCGAAGAACGT
TTTCCAATGATGAGCACTTTTAAAGTTCTGCTATGTGGCGCGGTATTATCCCGTGTTGACGCCGGGCAAGAGCAACTCGGTCGCCGCATACACTATT
CTCAGAATGACTTGGTTGAGTACTCACCAGTCACAGAAAAGCATCTTACGGATGGCATGACAGTAAGAGAATTATGCAGTGCTGCCATAACCATGAG
TGATAACACTGCGGCCAACTTACTTCTGACAACGATCGGAGGACCGAAGGAGCTAACCGCTTTTTTGCACAACATGGGGGATCATGTAACTCGCCTT
GATCGTTGGGAACCGGAGCTGAATGAAGCCATACCAAACGACGAGCGTGACACCACGATGCCTGTAGCAATGGCAACAACGTTGCGCAAACTATTAA
CTGGCGAACTACTTACTCTAGCTTCCCGGCAACAATTAATAGACTGGATGGAGGCGGATAAAGTTGCAGGACCACTTCTGCGCTCGGCCCTTCCGGC
TGGCTGGTTTATTGCTGATAAATCTGGAGCCGGTGAGCGTGGGTCTCGCGGTATCATTGCAGCACTGGGGCCAGATGGTAAGCCCTCCCGTATCGTA
GTTATCTACACGACGGGGAGTCAGGCAACTATGGATGAACGAAATAGACAGATCGCTGAGATAGGTGCCTCACTGATTAAGCATTGGTAACTGTCGA
GCTCGCTTGGACTCCTGTTGATAGATCCAGTAATGACCTCAGAACTCCATCTGGATTTGTTCAGAACGCTCGGTTGCCGCCGGGCGTTTTTTATTGG
TGAGAATCCAAGCACTAGTAACAACTTATATCGTATGGGGCTGACTTCAGGTGCTACATTTGAAGAGATAAATTGCACTGAAATCTAGTAATATTTT
ATCTGATTAATAAGATGATCTTCTTGAGATCGTTTTGGTCTGCGCGTAATCTCTTGCTCTGAAAACGAAAAAACCGCCTTGCAGGGCGGTTTTTCGA
AGGTTCTCTGAGCTACCAACTCTTTGAACCGAGGTAACTGGCTTGGAGGAGCGCAGTCACCAAAACTTGTCCTTTCAGTTTAGCCTTAACCGGCGCA
TGACTTCAAGACTAACTCCTCTAAATCAATTACCAGTGGCTGCTGCCAGTGGTGCTTTTGCATGTCTTTCCGGGTTGGACTCAAGACGATAGTTACC
GGATAAGGCGCAGCGGTCGGACTGAACGGGGGGTTCGTGCATACAGTCCAGCTTGGAGCGAACTGCCTACCCGGAACTGAGTGTCAGGCGTGGAATG
AGACAAACGCGGCCATAACAGCGGAATGACACCGGTAAACCGAAAGGCAGGAACAGGAGAGCGCACGAGGGAGCCGCCAGGGGGAAACGCCTGGTAT
CTTTATAGTCCTGTCGGGTTTCGCCACCACTGATTTGAGCGTCAGATTTCGTGATGCTTGTCAGGGGGGCGGAGCCTATGGAAAAACGGCTTTGCCG
CGGCCCTCTCACTTCCCTGTTAAGTATCTTCCTGGCATCTTCCAGGAAATCTCCGCCCCGTTCGTAAGCCATTTCCGCTCGCCGCAGTCGAACGACC
GAGCGTAGCGAGTCAGTGAGCGAGGAAGCGGAATATATCCCCTAGGTCTAGGGCGGCGGATTTGTCCTACTCAGGAGAGCGTTCACCGACAAACAAC
AGATAAAACGAAAGGCCCAGTCTTTCGACTGAGCCTTTCGTTTTATTTGATGCCTCTAGATTATTG

Supplementary references

1 Shan, G. *et al.* A sensitive class specific immunoassay for the detection of pyrethroid metabolites in human urine. *Chemical research in toxicology* **17**, 218-225, doi:10.1021/tx034220c (2004).

2 Salema, V. *et al.* Selection of single domain antibodies from immune libraries displayed on the surface of E. coli cells with two beta-domains of opposite topologies. *PloS one* **8**, e75126, doi:10.1371/journal.pone.0075126 (2013).

3 Kim, H.-J. *et al.* Isolation of Alpaca Anti-Hapten Heavy Chain Single Domain Antibodies for Development of Sensitive Immunoassay. *Analytical chemistry* **84**, 1165-1171, doi:10.1021/ac2030255 (2012).

4 Anderson, J. C. Registry of Standard Biological Parts [parts.igem.org]. (2009).

5 Salis, H. M. The ribosome binding site calculator. *Methods in enzymology* **498**, 19-42, doi:10.1016/B978-0-12-385120-8.00002-4 (2011).
